# Single-session gamified virtual reality exposure therapy for spider phobia vs. traditional exposure therapy: study protocol for a randomized controlled non-inferiority trial

**DOI:** 10.1186/s13063-016-1171-1

**Published:** 2016-02-02

**Authors:** Alexander Miloff, Philip Lindner, William Hamilton, Lena Reuterskiöld, Gerhard Andersson, Per Carlbring

**Affiliations:** Department of Psychology, Stockholm University, Frescati Hagväg 8, 106 91 Stockholm, Sweden; Department of Clinical Neuroscience, Karolinska Institutet, Stockholm, Sweden; Mimerse, Stockholm, Sweden; Department of Behavioral Sciences and Learning, Linköping University, Linköping, Sweden

**Keywords:** Specific phobia, Exposure therapy, Spiders, Virtual reality, Serious games, Gamification

## Abstract

**Background:**

Traditional one-session exposure therapy (OST) in which a patient is gradually exposed to feared stimuli for up to 3 h in a one-session format has been found effective for the treatment of specific phobias. However, many individuals with specific phobia are reluctant to seek help, and access to care is lacking due to logistic challenges of accessing, collecting, storing, and/or maintaining stimuli. Virtual reality (VR) exposure therapy may improve upon existing techniques by facilitating access, decreasing cost, and increasing acceptability and effectiveness. The aim of this study is to compare traditional OST with in vivo spiders and a human therapist with a newly developed single-session gamified VR exposure therapy application with modern VR hardware, virtual spiders, and a virtual therapist.

**Methods/design:**

Participants with specific phobia to spiders (*N* = 100) will be recruited from the general public, screened, and randomized to either VR exposure therapy (*n* = 50) or traditional OST (*n* = 50). A behavioral approach test using in vivo spiders will serve as the primary outcome measure. Secondary outcome measures will include spider phobia questionnaires and self-reported anxiety, depression, and quality of life. Outcomes will be assessed using a non-inferiority design at baseline and at 1, 12, and 52 weeks after treatment.

**Discussion:**

VR exposure therapy has previously been evaluated as a treatment for specific phobias, but there has been a lack of high-quality randomized controlled trials. A new generation of modern, consumer-ready VR devices is being released that are advancing existing technology and have the potential to improve clinical availability and treatment effectiveness. The VR medium is also particularly suitable for taking advantage of recent phobia treatment research emphasizing engagement and new learning, as opposed to physiological habituation. This study compares a market-ready, gamified VR spider phobia exposure application, delivered using consumer VR hardware, with the current gold standard treatment. Implications are discussed.

**Trial registration:**

ClinicalTrials.gov identifier NCT02533310. Registered on 25 August 2015.

## Background

Specific phobias are second only to major depression for lifetime prevalence of mental health disorders in the United States (16.6 % vs 15.6 %) [1]. The prevalence is somewhat reduced in older individuals (8.7 %) [2]. Estimates suggest 60–80 % of those with specific phobia are hesitant to seek help [3]. Some phobias are far more common than others [4], with half of reported phobias being fear of either heights or animals [5]. Spider phobia is among the most prevalent of animal phobias and is the most studied. The consequences of anxiety, worry, and avoidance behavior can have a large impact on quality of life, work, and leisure activities [6].

Exposure-based therapies in which an individual is systematically and repeatedly presented with a feared or avoided internal or external cue is highly effective in reducing anxiety disorders [7, 8]. Compared with imaginal exposure, evidence indicates that an in vivo (naturalistic setting) stimulus is the preferable exposure treatment method for specific phobia [6]. One-session therapy (OST) is considered the intervention of choice for in vivo specific phobia treatment in adults and children [9, 10]. OST sessions typically last up to 3 h and consist of graduated exposure to phobic stimuli, positive reinforcement, therapist modeling of non-phobic behavior, and cognitive restructuring of catastrophic beliefs [11].

The benefits of exposure therapy are limited by issues pertaining to both therapist and patient. Access to evidence-based treatments has been lacking [12], likely commensurate with the challenges of therapists locating appropriate material and/or stimuli, difficulties with conducting exposure work outside the clinic, and maintaining stimuli such as animals and insects. Even when these resources are available, participants may refuse to engage in therapy. Once explained, exposure therapy is turned down by 30 % of subjects [13], and this percentage is estimated to be significantly higher for OST if the endpoint is discussed beforehand [12]. Return of fear of the feared stimuli following treatment can also occur at a later date [14].

Virtual reality (VR) technology involving head-mounted, motion-tracked displays, accompanied by realistically rendered 3D computer-animated environments [15], offers researchers the opportunity to recreate phobic stimuli, manipulate and tailor key variables associated with stimulus presentation (color, size, and movement), context, scheduling, and intensity of exposure according to the patient’s needs, as well as to extract unprecedented amounts of data, such as gaze focus [16]. Previous generations of VR have already been used successfully in mental health treatments [17] for fear of flying, heights, public speaking, and spider phobia, among others [18]. VR thus has the potential to greatly increase accessibility and effectiveness of exposure treatments. In a survey of 777 undergraduate students who scored high in fear of spiders, more than 80 % expressed a preference for VR exposure treatment over in vivo treatment [19]. In recent studies, researchers have evaluated the unique opportunity of VR to inexpensively and relatively easily alter the context of exposure, an important moderator of treatment resurgence [20]. Participants treated using VR in multiple environments [21], and participants treated by exposure to spiders using video recordings of multiple areas of a house rather than a single area [22], were less likely to have return of fear following an aversive event.

Serious games designed for purposes other than entertainment and allowing users to experience situations impossible or dangerous in real life [23], with gamified elements such as points and goals to increase engagement [24], may provide a particularly promising advancement in exposure therapy. As reviewed by Botella et al. [25], game elements may reduce distress as compared with traditional exposure therapy [26]. Recommendations that patients continue to confront phobic stimuli posttreatment [12] may be facilitated by gamified VR content that can be played again and again [25].

The potential of VR notwithstanding, the quality of past VR exposure therapy research has historically been poor [27]. Well-designed randomized controlled trials are required before implementation in clinical practice can be recommended. Further, recent advances in VR technology have enabled unprecedented realistic stimuli to be rendered with less intrusive equipment, minimizing the risk of nausea and allowing longer sessions, though careful application design is still required [28]. A new generation of VR systems produced by some of the world’s largest technology companies, such as Sony (Project Morpheus), Microsoft (Hololens), HTC (Vive), and Facebook (Oculus Rift), promises to change VR from a professional niche product costing as much as $35,000 to a consumer product priced around $599 plus the cost of a competent computer [29]. Other developments in VR include the use of smartphone-based systems such as the Samsung Gear VR, used in the present study, the cost of which is negligible ($99 USD) if paired with a user’s preexisting smartphone [30]. From a research perspective, using VR can also reduce some of the complexity of carrying out exposure treatments, improve standardization of protocols and cost-effectiveness, and enable at-home self-care [31].

The parallel-group randomized controlled trial described in this protocol is designed to investigate non-inferiority of a novel, gamified VR OST program for spider phobia by comparison with traditional OST.

## Methods/design

This randomized controlled trial is registered in the ClinicalTrials.gov database (NCT02533310) and has received ethical approval from the Stockholm Regional Ethical Review Board (Dnr 472-31). Written informed consent will be obtained from all participants at the premeasurement occasion.

### Procedure

**Fig. 1:**
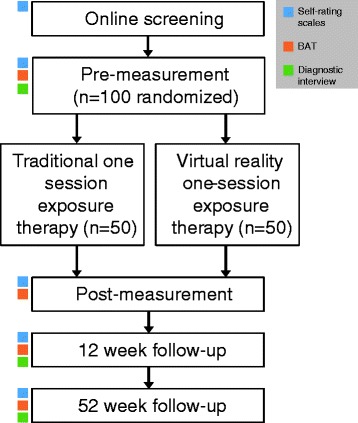
Study flowchart. *BAT* Behavioral Approach Test

To recruit a diverse sample, multiple recruitment methods will be used, primarily postings in online forums and through coverage in national television, newspapers, and magazines [32]. Potential participants will be directed to the study website (www.vimse.se), where they will find more information about the study and can complete the online screening battery. Participants meeting inclusion criteria will travel to Stockholm University to complete the premeasurement, including self-rating scales, a diagnostic interview, and a Behavioral Approach Test (BAT). If suitable for inclusion, participants will be randomized and, approximately 1 week after the premeasurement occasion, will complete the allocated treatment session. Posttreatment measurements will take place approximately 1 week after the treatment session. Additional follow-up measurements are planned after 12 and 52 weeks. See Fig. 1 for the study flowchart.

Subjects will be randomized to treatment arms (using a true random number generator: www.random.org) after completing the premeasurement in preset blocks of 4, 6, 8, or 10 (randomly sized) with even group allocation in each block. Randomization will be performed by a research assistant not otherwise involved in the study, and allocation will be blinded to all but the treatment coordinator and the assigned therapist. As in most psychological research, blinding participants to treatment allocation is not feasible. For ethical reasons, subjects are informed at premeasurement of the two possible treatment allocations, and their preference (and strength thereof) is noted to enable investigation of mediating effects of treatment preference. The therapist administering the posttreatment assessment will be blinded to treatment allocation until all outcome measures have been completed.

All data gathered during the study will be stored anonymously. No contractual agreements limit access by other investigators to this data or to final datasets, and upon completion the dataset generated in this trial will be published in a data repository (e.g., Dryad or figshare), accompanied by the script files to reproduce the statistical analyses.

### Sample

The sample will consist of 100 adults (age ≥18 years). To be included, participants need to reside in Sweden, be fluent in Swedish, and have the ability to travel to the study location on five separate occasions (pretest, treatment, posttest, and two follow-up occasions). Exclusion criteria include ongoing psychotherapy or psychotropic medication (unless on stable dosage for the previous 3 months and no changes planned during the study period), indications of suicidal ideation or other serious mental disorder (e.g., substance misuse, bipolar disorder, psychosis), and lack of stereoscopic vision or balance problems that would hinder experiencing the VR environment. To meet inclusion criteria, participants must score 9 or less on the BAT (see below) and meet the criteria set forth in the *Diagnostic and Statistical Manual of Mental Disorders, Fifth Edition* (DSM-5), for specific phobia, assessed using the Structured Clinical Interview for DSM Disorders [33] adapted for DSM-5 criteria [34].

### Measures

#### Primary outcome measure

Change from baseline on the BAT will serve as the primary outcome measure. To study the real-world effects of VR therapy, all participants will complete the same BAT featuring a real spider. The BAT [35] will feature 13 steps (scored 0–12) corresponding to sequentially closer contact with the spider. See Table 1 for sequence details. The BAT will begin with participants standing outside a room (approximately 3 × 5 m large) where there will be a table placed farthest away from the door, on which a transparent container (about 40 × 30 × 19 cm large) will house the spider (medium-sized; see below). The participants will be informed that the objective of the exercise is to pick up and hold the spider in their hands for 20 seconds. They will be encouraged to do their best but will be told that they can abort the exercise at any time. A pretreatment score of 9 or less [36] will be required for study inclusion.

**Table 1:** Behavioral Approach Test step specifications

Score	Details
0	Refusal to enter room
1	Enters room but stops before covering one-fifth of the distance to the container
2	Stops before covering two-fifths of the distance to the container
3	Stops before covering three-fifths of the distance to the container
4	Stops before covering four-fifths of the distance to the container
5	Stops before covering all of the distance to the container
6	Reaches the table with the container
7	Touches the container
8	Removes the lid of the container
9	Puts a hand inside the container
10	Touches the spider with at least one finger
11	Holds spider in hands for less than 20 seconds
12	Holds spider in hands for 20 seconds or more

#### Secondary outcome measures

In addition to the BAT, subjects will complete two self-report measures of fear toward spiders: the Spider Phobia Questionnaire (SPQ) [37] and the Fear of Spiders Questionnaire (FSQ) [38]. The Generalized Anxiety Disorder 7-item scale (GAD-7) [39] will be used to measure generic anxiety symptoms, and the Patient Health Questionnaire-9 (PHQ-9) [40] will be used to evaluate depression symptoms. Subjective quality of life will be measured with the Brunnsviken Brief Quality of life scale (BBQ) [41]. Meeting diagnostic criteria for specific phobia in the 12- and 52-week follow-up measurements will also serve as a secondary outcome measure. Swedish translations of all the self-rating scales will be used.

#### Other measures

At premeasurement, all participants complete the treatment Credibility/Expectancy Questionnaire (CEQ) [42] for both the traditional OST and VR treatments (in quasi-randomized order). Participants also complete the CEQ at postmeasurement with re-phrased items that ask respondents to evaluate their completed treatment. A number of additional surveys will be used to determine the influence of moderating factors on VR treatment outcome. Immediately following treatment, VR-treated participants will answer the Simulator Sickness Questionnaire (SSQ) [43], a measure of participant discomfort during treatment. At postmeasurement, VR-treated participants will further complete the iGroup Presence Questionnaire (IPQ) [44], a measure of the sense of actually being in the virtual environment, and the System Usability Scale (SUS) [45], which assesses product complexity and ease of use. At postmeasurement, all participants will complete the Adverse Effects Questionnaire, a novel measurement tool used to assess side effects of psychological treatments, as proposed by Rozenthal et al. [46]. Finally, at 12-week and 52-week follow-up, patients will answer questions regarding frequency and type of interactions with spiders since their last visit, as well as other forms of treatment received.

### Interventions

#### Traditional OST

Traditional OST treatment sessions will be held with participants on a one-to-one basis with clinical psychologists or clinical psychologists in their final year of training, all with past clinical cognitive behavioral therapy experience. All OST therapists will receive regular supervision from an experienced clinical psychologist and psychotherapist.

Traditional OST for spider phobia consists of a combination of gradated exposures to increasingly large, naturally occurring in vivo spiders and model learning of healthy, non-phobic behavior as demonstrated by the therapist [12]. Spiders used in traditional OST (and the BAT) will be harmless common varieties indigenous to the region, primarily of the genus *Tegenaria* (*T. domestica* and *T. atrica*). Spiders will be classified according to size: small (5–15 mm), medium (15–25 mm), or large (>25 mm).

The goal of OST is to bring patients a greater sense of control and a recognition that their feared catastrophic outcome will not occur (e.g., they will not have a heart attack). OST is performed for a maximum 3-h duration during a morning or afternoon. The patient is first explained the rationale and content of the treatment, then their catastrophic beliefs and safety behaviors are explored and avoidance activities are normalized. Participant modeling encourages careful observation by patients and that they not close their eyes or look away. Subjective units of distress (SUD) ratings are used to determine when to move on to a more challenging stage of treatment (100 % denotes the most anxiety-provoking situation experienced in relation to the phobia). Humor can be used to put the patient’s reactions into perspective and reduce the patient’s anxiety [47].

The initial graduated exposure step (featuring a small spider) teaches the patient to capture a spider with a glass with the intention of removing it from the building. The next exposure step has the patient touch various parts of the spider. Interactions with the spider are best organized as behavioral experiments in which the patient’s beliefs about what may occur are tested against reality. The exposure steps that follow have the spider crawl first on the patient’s hand then up to the elbow, and finally the spider is allowed to crawl on the patient’s body, from the knee to the waist and from the shirt up to the neck. These steps are then repeated using first a medium spider and then a large spider. The final stage has the patient handle two spiders, one in each hand, and an overlearning step can be added in which the spider crawls on the patient’s hair and cheek. The patient should have no or low anxiety in the last exposure stages and should no longer believe their catastrophic cognitions.

#### Virtual reality one-session therapy

**Fig. 2:**
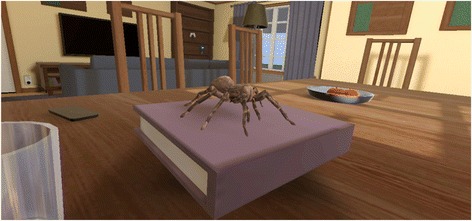
Screenshot from the virtual reality application

VR OST was designed as a serious game [23] with game progression entailing gradually increased stimuli intensity. The application consists of a number of zones, each containing three types of gamified tasks for the user to complete: (1) looking at spiders, (2) interacting with spiders to complete rudimentary game mechanics, and (3) a task where the user is approached by a spider. With completion of tasks and progression through the zones, spider stimuli become increasingly more intense, from a cute, cartoonish spider through realistically depicted tarantulas. See Fig. 2 for a screenshot from the application. Two virtual environments will be used: a living room environment and an outdoor suburban backyard environment. User input is strictly gaze-derived and is used for the interactive game mechanics, reporting SUD and more. Once initiated, the application is fully automated (i.e., no therapist action required) and includes a system for saving and compiling SUD and other input, a virtual therapist providing instructions through voiceover, support and summaries of progress, and information about spiders. Unlike the OST treatment, participants in the VR condition will be seated throughout the duration of therapy, but both groups will be time-limited to 3-h duration. Although the VR treatment is automated, a clinical psychologist will be in attendance (one-to-one) with the participant in case of technical or other difficulties.

In this study, the Samsung Gear VR platform (powered by a Samsung Galaxy Note 4 or Galaxy S6, both running Android 5.0) will be used to create the VR environment. Availability of the VR application on other VR platforms (e.g., Oculus and Vive) is planned.

### Analyses

Data will be analyzed on an intention-to-treat basis, using mixed effects models [48]. This trial was powered to detect a non-inferiority margin of a 2-point between-group difference, with a standard deviation of 4. The margin and standard deviation were based on previous research on traditional OST [36]. In accordance with the non-inferiority design, we hypothesize that the lower bound of the 95 % confidence interval of the between-group difference will not be larger than 2. On the basis of these parameters (and 80 % power), the study will require 50 participants assigned to each group (*N* = 100 total). In addition to the non-inferiority analyses, standard mixed models will be used to investigate effects of time and group on primary and secondary outcome measures. Within- and between-group effect sizes will be calculated using Cohen’s *d*. The influence on outcomes of demographic and other non-clinical variables (e.g., experience with gaming) will also be examined. All design, implementation, and reporting will be carried out in accordance with CONSORT and SPIRIT guidelines [49–51].

## Discussion

The aim of this study is to evaluate the effectiveness of VR exposure therapy compared with traditional one-session exposure therapy using a randomized controlled design and subjects diagnosed with spider phobia. The introduction of modern VR headsets combined with powerful but relatively inexpensive computers capable of displaying realistic stimuli, in addition to the historical lack of high-quality randomized controlled trials [52], suggest that this is an opportune time to make use of this rapidly advancing technology and translate it into clinically validated mental health treatments. VR exposure therapy has already been theoretically and empirically evaluated to be well-suited for the treatment of specific phobias [53]. The authors of a recent meta-analysis [54] provided additional evidence based on behavioral assessment rather than self-report of internal states. VR exposure-treated subjects, in their review of 14 studies, improved significantly after treatment (*g* = 1.23) and compared with control subjects (*g* = 1.41). No significant differences were identified after treatment and in follow-up between in vivo and VR exposures.

Conducting VR exposure therapy using modern, commercially available VR equipment may prove even more powerful. In addition to the evaluation of modern VR hardware, our intent in this study is also to evaluate a newly developed, gamified exposure application for the treatment of specific phobias. The software includes advancements such as multiple open-ended exposure scenarios relying on gamification to improve engagement and interest; multiple stimulus intensity variables such as appearance (small to large spiders, cartoon-like to hairy), behavior (more or less predictable, static, and aggressive), a variable number of spiders, and changes in lighting and protective barriers (such as caged or not); a virtual therapist that guides the participant in the use of the application and provides psychoeducation and expert advice about spiders; and inclusion of a gaze direction trackpad to allow participants to interact with the stimuli.

Recent evidence indicates that treatment benefits of exposure therapy accrue as a result of new inhibitory learning and not habituation of the conditioned response to phobic stimuli [55]. Physiological habituation (viz., reduced heart rate and galvanic skin response) may serve as a safety signal alleviating fear in the short term but inhibiting long-term learning and extinction [56]. Serious game treatments in which there is a sense of play, interactivity, flow, and creative solutions have the potential to promote new learning [57]. In an exposure therapy context, these should create engagement (rather than avoidance) by producing a mismatch between expectancy and outcome and require flexible responding [55]. In a study in which a fear of heights group received random levels of exposure intensity and approached stimuli in multiple ways versus a steady intensity increase group, researchers found improved fear reduction in the former, without a need for physiological habituation [58].

Limitations of the present study include an exposure application that involves multiple unique design specifications, making disentangling of specific therapeutic factors difficult. Despite the complete automation of the VR application, psychologists will be in attendance to resolve technical difficulties and ensure treatment compliance and will be present to assist patients if needed. Future studies will benefit from having the patient administer VR treatment independently to ensure true isolation of treatment effects. In addition, physiological monitoring of subjects will not be possible during this study but may prove helpful in interpreting the results of future studies.

In sum, spider phobia is a common disorder [1] that has a negative impact on life, work, and leisure activities [6] but goes largely untreated [3]. VR exposure therapy may provide improvements in efficacy [15], access, standardization of protocols, and cost-effectiveness [31]. Although not evaluated in this study, virtual OST also has the potential to conveniently continue maintenance therapy once regular treatment has been completed. This study is, to our knowledge, the first to test the efficacy of a modern, market-ready VR application for the treatment of spider phobia, and it will assist in the development of a new method for the delivery of evidence-based treatments.

## Trial status

At time of initial manuscript submission (August 2015), recruitment was ongoing. Enrolment and the active treatment period are expected to be completed in January 2016.

## Abbreviations

BAT: Behavioral Approach Test
BBQ: Brunnsviken Brief Quality
CEQ: Credibility/Expectancy Questionnaire
DSM: *Diagnostic and Statistical Manual of Mental Disorders*
FSQ: Fear of Spiders Questionnaire
GAD-7: Generalized Anxiety Disorder 7
IPQ: iGroup Presence Questionnaire
OST: one-session therapy
PHQ-9: Patient Health Questionnaire-9
SPQ: Spider Phobia Questionnaire
SSQ: Simulator Sickness Questionnaire
SUD: subjective units of distress
SUS: System Usability Scale
VR: virtual reality
